# Hepatic Cholesterol-25-Hydroxylase Overexpression Improves Systemic Insulin Sensitivity in Mice

**DOI:** 10.1155/2017/4108768

**Published:** 2017-02-19

**Authors:** Britta Noebauer, Alexander Jais, Jelena Todoric, Klaus Gossens, Hedwig Sutterlüty-Fall, Elisa Einwallner

**Affiliations:** ^1^Department of Laboratory Medicine, Medical University of Vienna, Vienna, Austria; ^2^Laboratory of Gene Regulation and Signal Transduction, Departments of Pharmacology and Pathology, UCSD School of Medicine, San Diego, CA, USA; ^3^Max Planck Institute for Immunobiology and Epigenetics, Freiburg, Germany; ^4^Institute of Cancer Research, Department of Medicine I, Comprehensive Cancer Center, Medical University of Vienna, Vienna, Austria

## Abstract

Obesity is a major risk factor for several diseases including diabetes, heart disease, and some forms of cancer and due to its rapidly increasing prevalence it has become one of the biggest problems medicine is facing today. All the more surprising, a substantial percentage of obese patients are metabolically healthy when classified based on insulin resistance and systemic inflammation. Oxysterols are naturally occurring molecules that play important role in various metabolic and inflammatory processes and their levels are elevated in patients suffering from obesity and diabetes. 25-Hydroxycholesterol (25-OHC) is produced in cells from cholesterol by the enzyme cholesterol 25-hydroxylase (Ch25h) and is involved in lipid metabolism, inflammatory processes, and cell proliferation. Here, we investigated the role of hepatic Ch25h in the transition from metabolically healthy obesity to insulin resistance and diabetes. Using several different experimental approaches, we demonstrated the significance of Ch25h on the border of “healthy” and “diseased” states of obesity. Adenovirus-mediated Ch25h overexpression in mice improved glucose tolerance and insulin sensitivity and lowered HOMA-IR. Our data suggest that low hepatic Ch25h levels could be considered a risk marker for unhealthy obesity.

## 1. Introduction

Obesity has become a major global health burden due to rapid increase in prevalence that has more than doubled since 1980 (WHO, 2014). There are over 1.9 billion overweight adults worldwide and more than 600 million of them are classified as obese. Obesity has been officially recognized as a disease by American Medical Association and it poses one of the main risk factors for chronic diseases such as type 2 diabetes, heart disease, hypertension, osteoarthritis, sleep apnea, and several types of cancer [1, 2]. In addition, obesity is a part of metabolic syndrome, which is a group of linked disorders including elevated blood pressure, fasting plasma glucose, and serum triglycerides and low high-density lipoprotein (HDL) levels, associated with increased risk of heart disease and type 2 diabetes [3]. Interestingly, up to one out of four obese individuals is metabolically healthy [4] classified on the basis of indices for insulin resistance and systemic inflammation.

Factors discriminating between the “healthy” and the “unhealthy” obese individuals are still ill-defined. Metabolically healthy obesity is associated with smaller adipocyte size and higher ratio of subcutaneous versus visceral fat depots [5]. The amount of visceral fat also correlates with intrahepatic triglyceride content [6–8]. In line with this, impaired insulin action is linked to accumulation of saturated fatty acids and ectopic lipids in the liver [9]. Increased hepatic lipogenesis is one of the hallmarks of many obesity and diabetes models [10] and hepatic steatosis might be a major mechanism for developing insulin resistance [11–13]. The relationship between lipid accumulation in various tissues and insulin resistance still needs further studies; however, it is possible that ectopic triglycerides in liver are the primary cause for metabolic abnormalities that accompany “unhealthy” obesity.

Oxygenated derivatives of cholesterol, oxysterols, are elevated in patients suffering from obesity, diabetes, hypercholesterolemia, and atherosclerosis [14–16]. The central role of oxysterols is the regulation of cholesterol metabolism, which is controlled, in part, by their binding to nuclear receptors (NRs), such as liver X receptors (LXRs) and retinoid-related orphan receptors RORs [17, 18]. The activation of LXRs induces a range of genes that are involved in cholesterol transport and excretion and encode anti-inflammatory proteins [19–21]. In general, when cells are challenged with excess cholesterol, LXR-dependent transcriptional signaling enables them to reconstitute cholesterol homeostasis [21]. In addition, LXRs have been shown to suppress immune responses mediated by macrophages and other immune cells [22, 23]. However, at the same time LXRs are able to directly activate lipogenic genes and stimulate de novo lipogenesis [20, 24] which can result in hepatic steatosis and insulin resistance [25]. There are several naturally occurring oxysterols including the three most prominent ones, 25-hydroxycholesterol (25-OHC), 24-hydroxycholesterol, and 27-hydroxycholesterol. 25-OHC has been shown to be a regulator of lipid metabolism, inflammatory processes, and cell proliferation [26]. Beyond its role in regulation of lipid metabolism 25-OHC can serve both pro- and anti-inflammatory functions [27, 28]. The formation of 25-OHC is catalyzed by the enzyme cholesterol 25-hydroxylase (Ch25h) [29, 30]. Most tissues show very low Ch25h expression levels [31]; however in vivo treatment with Toll-like receptor (TLR) agonists increases expression, most notably in liver and macrophages that are a potent source of inducible Ch25h [32, 33]. Here we show that Ch25h levels are decreased in obese insulin resistant compared to obese insulin sensitive mice and an increase in hepatic Ch25h mRNA expression and protein levels improves insulin sensitivity, which makes it an important factor in the transition from “healthy” to “diseased” obesity.

## 2. Materials and Methods

### 2.1. Reagents

All chemicals and reagents were obtained from Sigma, unless stated otherwise.

### 2.2. qPCR

Quantitative PCR analysis was conducted as previously described [34]. Total RNA was extracted from tissues and cells using RNA isolation kits (RNeasy, QIAGEN; TRIzol, Invitrogen). The isolated total RNA was converted into cDNA via reverse transcription using commercially available kits (High-Capacity cDNA Reverse Transcription Kit, Applied Biosystems). iQ SYBR Green Supermix (Bio-Rad Laboratories) was used for qPCR reactions. Primer-only controls were included for ensuring the absence of primer dimers and a check for unspecific products was conducted by performing a postamplification melting curve analysis. Normalization was done by normalizing threshold cycles (Ct values) to acidic ribosomal phosphoprotein P0 (Rplp0) within each sample for obtaining ΔCt values sample-specific ( = Ct  gene  of  interest − Ct  Rplp0). Fold expression levels were obtained by calculating 2^−ΔΔCt^ levels (ΔΔCt = ΔCt  treatment − ΔCt  control).

### 2.3. Western Blot

Tissues were homogenized in RIPA buffer (0.5% NP-40, 0.1% sodium deoxycholate, 150 mM NaCl, and 50 mM Tris-HCl, pH 7.5) containing protease inhibitors (Complete Mini, Roche). Homogenate was cleared by centrifugation at 4°C for 30 min at 15,000 ×g, following the recovery of the supernatant containing the protein fraction. The BCA Protein Assay Kit (Pierce) was used for determination of protein concentration in the supernatant. SDS-PAGE resolving of 20 *µ*g of protein was followed by transfer to PVDF membranes (GE Healthcare). 5% BSA in Tris-buffered saline containing 0.2% Tween-20 (TBS-T) was used for blocking membranes, which were then incubated with primary antibodies at 4°C overnight. The following antibodies were used: anti-*β*-actin (1 : 500; A5441, Sigma) and anti-CH25H antibody (1 : 1000 ab76478, Abcam). Incubated membranes were washed and probed with appropriate secondary antibody (NA 934, anti-rabbit IgG, 1 : 20,000; NA 931, anti-mouse IgG, 1 : 20,000; GE Healthcare). Detection of antigen-specific binding of antibodies was performed with SuperSignal West Femto and Pico Kits (Pierce) by the use of a ChemiDoc XRS Imager (Bio-Rad). Image Lab Software version 3.0.1 was used for Image analysis (Bio-Rad).

### 2.4. 25-Hydroxycholesterol ELISA

Liver samples were homogenized and run along with standards supplied with the kit as per the user manual supplied with 25-hydroxycholesterol ELISA kit (#B0344, Glory Science). Optical density (OD) was measured at 450 nm.

### 2.5. Animals

Male C57BL/6J mice (wild type, wt) were purchased from the Charles River Laboratories (Sulzfeld, Germany). All mice were maintained on a 12-hour light/dark cycle and had free access to food and water. Mice were sacrificed by cervical dislocation and liver samples were collected and immediately snap-frozen in liquid nitrogen. All animal procedures used in this study were reviewed and approved by the Animal Care and Use Committee of the Medical University of Vienna and conducted according to FELASA guidelines. At 6 weeks of age, mice were placed for 24 weeks on high-fat (60% fat calories, D12492, Research Diets Inc., New Brunswick, NJ, USA; *n* = 10/group) and low-fat (10% fat calories, D12450B, Research Diets Inc.; *n* = 10/group) diet to induce obesity and to serve as lean controls. Liver protein samples were collected from lean and obese C57BL/6J mice for further experiments.

### 2.6. Oral Glucose Tolerance Test

Following an overnight fast, mice were given glucose (1 g/kg) via oral gavage. At the indicated time points, blood samples for determination of glucose and insulin levels were collected from the tail vein. An Accu-Chek (Roche) glucometer was used for assessing glycemia. Levels of plasma insulin were determined by the use of Ultrasensitive Mouse Insulin ELISA kit (Mercodia).

### 2.7. Insulin Tolerance Test

Following a 2-hour fast, insulin tolerance was assessed by the intraperitoneal administration of regular human insulin (0.75 U/kg Actrapid; Novo Nordisk) and monitoring blood glucose. Hepatic insulin sensitivity was assessed in adenovirus-injected mice by performing low-dose insulin tolerance tests (0.1 U/kg).

### 2.8. Obese Insulin Sensitive (obIS) and Obese Insulin Resistant (obIR) C57BL/6J Mice

As previously described [35], we generated a large cohort of high-fat diet- (HFD-) treated male C57BL/6J mice and stratified them according to glucose and insulin tolerance. Subgroups were selected by exhibiting either insulin sensitivity (IS, *n* = 5/group) or insulin resistance (IR, *n* = 5/group) despite comparable weight gain on the HFD. Liver samples were collected from these obese insulin sensitive (obIS) and obese insulin resistant (obIR) C57BL/6J mice fed for further analyses.

### 2.9. Adenovirus Experiments

Adenovirus experiments targeting livers were conducted with in vivo grade CH25H (AdCH25H) and LacZ (AdLacZ) expressing viruses, produced according to published protocols [36]. FuGENE 6 Transfection Reagent (Roche) was used for cotransfecting Sfi I-digested Adlox plasmid DNA with psi5 DNA into Cre8 cells. Cells were collected by centrifugation, three days after transfection, and the recombined viruses were extracted from cell pellets by four cycles of freeze and thaw. Centrifugation was used to remove cell debris. When selecting for the recombined adenoviruses, we performed seven reinfection cycles in Cre8 cells. Adenoviral particles were amplified by the use of HEK293 cells (ATCC). Purification of amplified AdCH25H and AdLacZ was achieved by chloride density-gradient ultracentrifugation. They were then collected from the gradient, diluted in 2x storage buffer 1 : 1 (10 mM Tris pH 8.0, 100 mM NaCl, 0.1% BSA, and 50% glycerol), and afterwards stored at −20°C in small aliquots. The in vitro tail vein injections were implemented as previously described [37]. As recommended, a total volume of 200 *µ*l of AdHO-1 and AdLacZ particles diluted with PBS (0.2 × 10^9^ pfu/g bodyweight) was injected into the tail vein of the respective recipient mice. After day 5 and day 7 of postinjection, OGTTs and ITTs were performed.

## 3. Results

### 3.1. Liver Ch25h Expression Is Decreased during HFD

**Figure 1:**
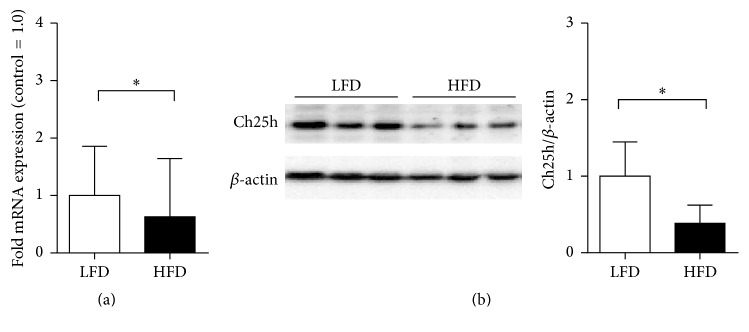
HFD treatment on mice resulted in decreased hepatic Ch25h protein levels. (a) Ch25h mRNA levels in isolated livers obtained from mice on HFD and control LFD. mRNA levels of hepatic Ch25h levels in LFD mice were xxx-fold higher comparing to mice on HFD. ^*∗*^*p* = 0.036, *n* = 10 per group. (b) Immunoblot analysis of Ch25h protein in isolated livers obtained from mice on HFD and control LFD. Mice on HFD showed a clear reduction in liver Ch25h protein levels comparing to the littermate controls. *β*-Actin was used as a loading control. ^*∗*^*p* = 0.046, *n* = 3 per group.

It has been shown that Ch25h is involved in the regulation of lipid metabolism; therefore our first aim was to investigate the role of hepatic Ch25h levels in obesity. We challenged C57BL/6J mice with high-fat diet (HFD) and observed a clear reduction of Ch25h mRNA and protein levels compared to controls in the isolated livers of mice on HFD treatment (Figures 1(a) and 1(b)). Recently, contrary to our data, Guillemot-Legris and colleagues indicated that the levels of hepatic 25-hydroxycholesterol were not affected during a HFD treatment in mice [38]. This led us to the search for published human datasets on CH25H levels in obesity.

### 3.2. CH25H Levels Decrease in Livers of Obese Subjects but Not in Livers of Diabetic Patients

**Figure 2:**
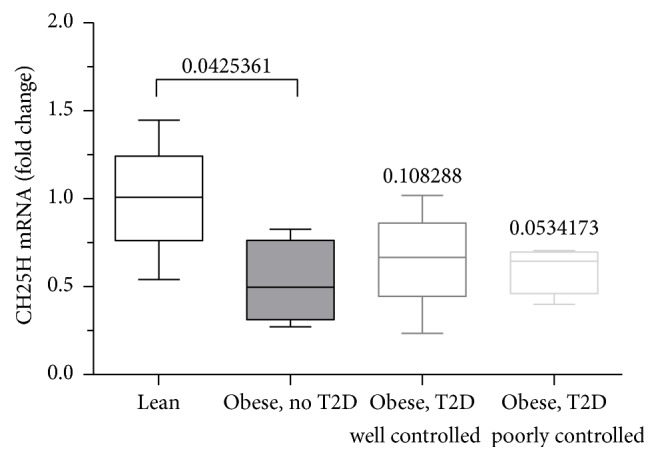
Dataset on liver CH25H mRNA levels in lean subjects and obese patients with and without type 2 diabetes. CH25H mRNA levels are decreased in obese patients without diabetes versus lean subjects. Levels of obese patients showing poorly controlled type 2 diabetes patients are decreased as well whereas *p* values for mRNA levels of obese, well controlled type 2 diabetes patients are not significant. GDS3876/206932_at/CH25H. Obese patients with and without type 2 diabetes: liver.* Homo sapiens*.

We performed an unbiased search for human datasets and identified one human liver dataset (NCBI, GEO dataset GDS3876), comparing lean and obese patients with and without type 2 diabetes. In humans again, CH25H levels in liver samples are consistently lower in obese patients. The data shows a tendency of lower CH25H levels between only obese and obese patients with type 2 diabetes poorly controlled whereas this effect was lost in obese patients with type 2 diabetes mellitus when well controlled (Figure 2). This led us to further investigate the role of hepatic CH25H levels in insulin sensitivity.

### 3.3. Ch25h Protein Levels Are Decreased in Insulin Resistant Mice

Exploring the link between Ch25h and “healthy” versus “diseased” states of obesity, we measured hepatic Ch25h levels in two cohorts of male C57BL/6J mice that were fed HFD and stratified according to their response to oral glucose and insulin tolerance tests [35].

**Figure 3:**
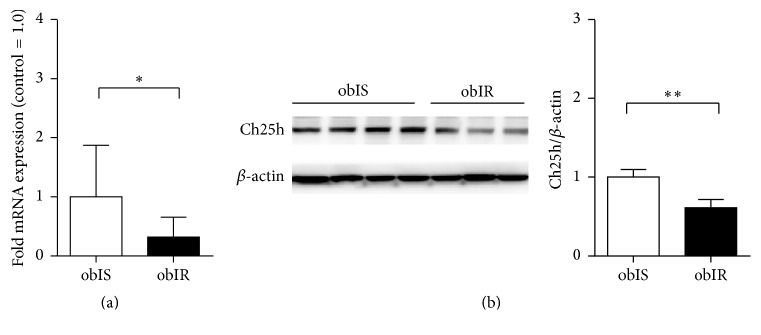
Obese IS mice show higher hepatic Ch25h levels than obese IR mice. (a) Relative Ch25h levels in isolated livers obtained from obese IR and obese IS C57BL/6J mice after 16 weeks on HFD. mRNA levels of hepatic Ch25h levels in obese IS mice were significantly higher comparing to obese IS mice. ^*∗*^*p* = 0.020, *n* = 5 per group. (b) Immunoblot analysis of Ch25h protein in isolated livers obtained from obese IR and obese IS C57BL/6J mice after 16 weeks on HFD. For immunoblot *β*-actin was used as a loading control. ^*∗∗*^*p* = 0.002, *n* = 3-4 per group.

**Figure 4:**
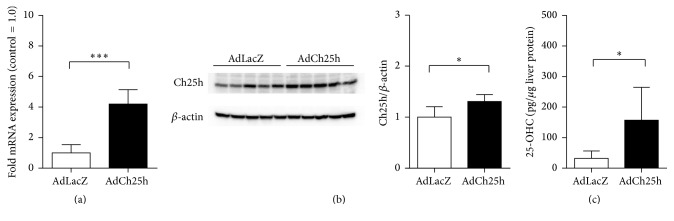
Tail vein injection of adenoviral* Ch25h* showed a successful hepatic* Ch25h* and 25-hydroxycholesterol overexpression. (a) Relative Ch25h levels in AdLacZ and AdCh25h transduced livers. mRNA levels of hepatic Ch25h levels increased 5-fold compared to AdLacZ injected controls. ^*∗∗∗*^*p* < 0.001, *n* = 5 per group. (b) Ch25h immunoblot of AdLacZ and AdCh25h transduced liver biopsies. Ch25h protein levels are clearly increased in livers of AdCh25h treated mice compared to AdLacZ transduced controls. *β*-Actin was used as a loading control. ^*∗*^*p* = 0.034, *n* = 5 per group. (c) 25-OHC levels in AdLacZ and AdCh25h transduced livers. 25-OHC levels are significantly increased in livers of AdCh25h treated mice compared to AdLacZ transduced controls. ^*∗*^*p* = 0.035, *n* = 5 per group.

Interestingly, our results showed a notable decrease in the levels of hepatic Ch25h mRNA of the mice belonging to the obese insulin resistant subpopulation (Figures 3(a) and 3(b)). Therefore, Ch25h shows significant predictive power in distinguishing these two groups and could be considered as a marker of metabolically diseased obesity. Since hepatic Ch25h was downregulated in mice treated with high-fat diet, in line with published human dataset as shown in Figure 2, in order to corroborate this finding we performed the overexpression of hepatic Ch25h via tail vein injection of adenoviral Ch25h under basal conditions (AdCh25h). Successful upregulation of Ch25h mRNA and protein levels is shown in Figures 4(a) and 4(b), respectively. Adeno-LacZ transduced mice served as control. To examine whether the upregulation of Ch25h protein directly influences the levels of 25-hydroxycholesterol (25-OHC) in hepatic tissue, we measured 25-OHC levels in liver biopsies of AdCh25h and AdLacZ treated mice (Figure 4(c)). 25-OHC levels were significantly elevated in the AdCh25h group compared to controls.

**Figure 5:**
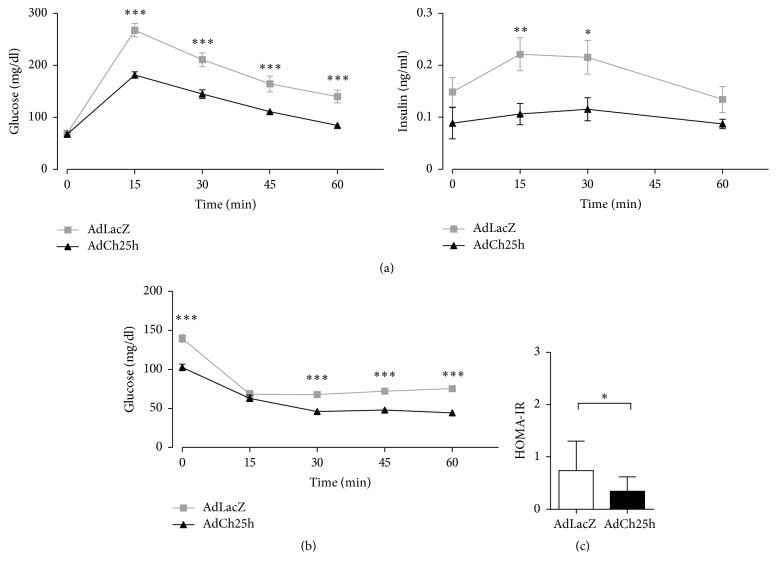
Overexpression of hepatic Ch25h improved glucose tolerance, insulin sensitivity, and lowered HOMA-IR. (a) Oral glucose tolerance tests (OGTT) performed in AdLacZ and AdCh25h transduced C57BL/6J animals. The glucose (mg/dl) and insulin (ng/ml) levels were measured 15, 30, 45, and 60 min after administering glucose solution (1 g/kg). Blood glucose and insulin levels were significantly lower in the AdCh25h transduced subpopulation. Two-sided *p* values obtained from unpaired *t*-test. Results are mean ± SEM. ^*∗*^*p* < 0.05, ^*∗∗*^*p* < 0.01, and ^*∗∗∗*^*p* < 0.001. *n* = 5 per group. (b) Insulin tolerance tests (ITTs) on AdCh25h and AdLacZ transduced C57BL/6J mice. Regular human insulin was administered intraperitoneally and blood glucose levels were monitored and measured 15, 30, 45, and 60 min after injections. The blood glucose levels were clearly decreased in mice treated with AdCh25h. Two-sided *p* values obtained from unpaired *t*-test. Results are mean ± SEM. ^*∗∗∗*^*p* < 0.001, *n* = 5 per group. (c) HOMA-IR levels of AdLacZ and AdCh25h transduced C57BL/6J mice. Comparison of the control group to the AdCh25h treated mice showed a significant decrease in HOMA-IR levels. Results are mean ± SEM. ^*∗*^*p* < 0.05, *n* = 5 per group.

Importantly, Ch25h overexpression in normal chow-fed C57BL/6J mice further improved glucose tolerance, insulin sensitivity, and lowered HOMA-IR relative to Adeno-LacZ injected controls (Figures 5(a)–5(c)). This in vivo gain-of-function study indicates elevated hepatic Ch25h levels are able to improve insulin sensitivity and therefore induce a metabolically healthy form of obesity.

## 4. Discussion

The idea that oxysterols are general regulators of lipid metabolism is not new, since evidence exists for their activating or repressing interactions with NRs [18–20, 39]. 24S-Hydroxycholesterol (24-OHC) levels are involved in regulating cholesterol homeostasis in the brain [40] and were found to play a role in neurodegenerative diseases [41–46]. Another recent study by Guillemot-Legris and colleagues [38] observed not only a decrease of 4*β*-OHC in the plasma of human obese patients, but also the fact that the levels in the liver, hypothalamus, and adipose tissue were significantly lowered. Literature pointed out a metabolic role of 27- and 25-hydroxycholesterol in the liver, the key regulator of cholesterol metabolism in an organism. Administration of 27-OHC can reduce hepatic inflammation and regulate the intracellular distribution of cholesterol [47–49]. Inflammation is one of the key events that triggers insulin resistance and subsequently hyperglycemia in type 2 diabetes [50]. Reboldi and colleagues identified Ch25h as a crucial part in the negative feedback mechanism that regulates production of IL-1 family cytokines during inflammatory processes which involve type I IFN [51]. They also showed that 25-HC has a repressive effect on Il1b expression and is a broad inhibitor of inflammasome activity. When they tested the impact of Ch25h-deficiency on serum levels of inflammatory markers, they observed that Ch25h-deficient mice showed higher levels of IL-1*α*, IL-1*β*, and IL-18 upon LPS-treatment, compared to controls [51]. This is in line with our observations that insulin resistant obese mice showed a significant decrease in Ch25h mRNA and protein levels in the liver, compared to their metabolically healthy obese littermates. Supporting these findings in the context of obesity and diabetes, Osborn and colleagues observed significantly reduced HbA1c levels when they treated mice suffering from T2D with anti-IL-1*β* antibodies, which indicates a beneficial effect on glucose tolerance and/or insulin resistance [52]. The role of inflammasomes in metabolic disorders, including obesity and diabetes, has been extensively studied over the last years. There are several reported inflammasomes, but one of the most prominent is the NLRP3 inflammasome, which has been shown to have a very broad range of targets [53]. NLRP3-deficient mice have been shown to have improved glucose tolerance and insulin sensitivity [54]. Similar to these findings, Wen and colleagues have studied the effects of saturated versus unsaturated fatty acids on the release of chemokines and the NLRP3 inflammasome activity. They could show that the saturated fatty acid, whose plasma concentration is clearly elevated after a HFD, has a trigger role in the NLRP3-dependent release of IL-1*β* and IL-18 and prevents regular insulin signaling in target tissues eventuating in insulin resistance, whereas administering unsaturated fatty acids had no influence on inflammasome activation or IL-1*β* release [55]. Collectively, literature indicates the activation of inflammasomes and release of IL-1*β* chemokines as pivotal underlying factors in the development of insulin resistance, which could be an explanation why the overexpression of Ch25h resulted in lower glucose and insulin levels and improved insulin sensitivity.

On the other hand, data exist showing that 25-HC can amplify secretion of inflammatory cytokines such as macrophage colony-stimulating factor (M-CSF), IL-6, and IL-8 [56, 57]. These at first sight contradicting results indicate that 25-HC cannot only be seen as a positive or negative regulator of the immune system. Wunderlich and colleagues have shown that IL-6 action, which originates from the activation of Kupffer cells in the liver, leads to a restricted inflammation, not only locally but also systemically, and therefore prevents insulin resistance [58]. Therefore, previously published data on 25-HC having an amplifying effect on the secretion of IL-6 are in line with our results showing that overexpression of Ch25h was beneficial for further improving glucose tolerance, insulin sensitivity, and high HOMA-IR levels. As these different aspects of inflammatory processes in the development of insulin resistance and their correlation with elevated hepatic levels of Ch25h in mice could be explained with both attempts, based on already published data, further investigation is needed for elucidating the direct mechanisms and pathways responsible for our observed effects.

Recently, Tuong and colleagues reported that a siRNA mediated knock-down targeting Ch25h resulted in a significant increase in lipid droplets (LDs) in bone marrow-derived macrophages (BMMs) [59]. Disorders of LDs, the major storage place for cholesterol, are associated with abnormalities in lipid trafficking that are closely associated with obesity, increased inflammatory responses, and insulin resistance. Converting cholesterol in hydroxycholesterol, or in this case 25-HC, might be one of the mechanisms that cells use to rebalance excess amounts of cholesterol. This could explain why we observed low hepatic Ch25h levels in both insulin sensitive and insulin resistant obese mice.

To our knowledge, no reports are available on precise comparisons of Ch25h expression levels in the livers of obese insulin sensitive and insulin resistant subjects or mice. Our findings redefine the current view of this undervalued cholesterol hydroxylase enzyme and indicate a potent role of Ch25h in the regulation of the whole-body metabolism. Our data are also indicative of Ch25h expression where not only is it downregulated in the wake of obesity, but also its importance is mainly at the point of decision between insulin resistance and insulin sensitivity. Further studies are necessary regarding the exact mechanisms of hepatic Ch25h action in the development of insulin resistance and subsequently type 2 diabetes.
